# Work Absenteeism in Inflammatory Bowel Disease Patients Related to Patient-Reported Anxiety Levels and Disease Activity: The IBD-GO-WORK Study

**DOI:** 10.3390/jcm14134410

**Published:** 2025-06-20

**Authors:** Raffaele Pellegrino, Ilaria De Costanzo, Giuseppe Imperio, Michele Izzo, Fabio Landa, Andrea Durante, Alessandro Federico, Antonietta Gerarda Gravina

**Affiliations:** 1 Hepatogastroenterology Division, Department of Precision Medicine, University of Campania Luigi Vanvitelli, Via L. de Crecchio, 80138 Naples, Italy; 2 Occupational Health and Hygiene Service, Local Health Authority of Naples 3 South, Via Marconi, 80059 Torre del Greco, Italy

**Keywords:** work absenteeism, HPQ, work productivity, patient-reported outcome

## Abstract

**Background/Objectives**: Patients with inflammatory bowel disease (IBD), whether affected by Crohn’s disease (CD) or ulcerative colitis (UC), are burdened by disability and a reduced quality of life. The individual’s regular participation in daily working life is a key factor among its determinants. This work aims to quantify work absenteeism in patients with IBD, profiling it concerning specific demographic variables, the degree of disease activity, and the level of self-reported anxious symptoms. **Methods**: A cross-sectional observational study targeted patients with a known diagnosis of IBD with disease activity no greater than moderate who were either employed or engaged in regular student activities. Participants were administered the Beck Anxiety Inventory (BAI) for the assessment of anxious symptoms, the Patient-Reported Outcome 2 (PRO-2) for evaluating IBD disease activity, and the Health and Work Performance Questionnaire (HPQ) short form for the analysis of work absenteeism, measured both as absolute and relative over two time frames (the last 7 days and the last 4 weeks). Within the HPQ, Likert scale (0–10) questions were administered to assess self-perceived work productivity. **Results**: A total of 300 patients were included [median age 43.5 years, IBD (UC 55.7%, CD 44.3%, sex (males 54%, females 46%)], recording absolute absenteeism of 56 (36–76) and 2 (−8–20) hours lost over 4 weeks and 7 days, respectively. The factors associated with worse absolute and relative absenteeism (both at 7-days and 4-weeks) were having CD (*p* < 0.001), having previous surgery (*p* < 0.05), and, exclusively in the 4-week assessment, being female (*p* < 0.05) and a smoker (*p* < 0.05). The BAI demonstrated a moderate correlation with 4-week absolute absenteeism (*ρ* = 0.374, *p* < 0.001), progressively increasing with anxiety severity. Additionally, the BAI was an independent predictor of a 25% work productivity loss over 4 weeks (aOR: 1.1, 95% CI 1.06–1.142, β = 0.096, *p* < 0.001). Disease activity measured based on PRO-2 strongly correlated with 4-week (*ρ* = 0.53, *p* < 0.001) and 7-day (*ρ* = 0.47, *p* < 0.001) absolute absenteeism. **Conclusions**: In conclusion, work absenteeism in IBD patients may be driven by the IBD phenotype, sex, anxiety, and disease activity. Improving these parameters could enhance productivity.

## 1. Introduction

Inflammatory bowel diseases (IBDs) are chronic immune-mediated conditions characterised by a chronic, relapsing-remitting, and self-sustained gastrointestinal inflammatory burden over time, which exposes the patient to a reduced quality of life and the development of short- and long-term complications (including neoplastic ones) and are associated with disability [1,2]. Moreover, IBDs, as is well known, are characterised by a highly varied constellation of symptoms, such as diarrhoea and rectal bleeding, abdominal pain, fatigue, and even extremely severe and life-threatening conditions, such as toxic megacolon and severe anaemia [3]. Additionally, there are specific features in patients with Crohn’s disease (CD), due to the transmural, potentially stenosing, and ubiquitous nature of the lesions throughout the gastrointestinal tract, such as malabsorption, fistulisation, and possible subocclusive or occlusive episodes [3]. In contrast, in patients with ulcerative colitis (UC), bowel urgency and the potential development of severe conditions, such as acute severe UC, represent distinctive characteristics [3]. It should also be noted that patients with IBD may present with extraintestinal manifestations (e.g., articular, cutaneous, ocular), which further contribute to the burden of disability in these patients [3].

Regular working life is fundamental to a normal quality of life, and the current consensus on therapeutic targets for IBD is increasingly raising the bar, moving from mere disease activity control to achieving a quality of life that overlaps with that of the general population (a so-called disease clearance) [4,5].

Patients with IBD have been described as experiencing losses in multiple determinants of regular quality of life, including mental health [6], genitourinary impairment [7,8], and regular physical activity [9,10]. Being a systemic disease, IBD is also associated with the wide-ranging impact of extra-intestinal manifestations [11,12].

All of this has been exacerbated by the recent COVID-19 pandemic, which has intensified all these social impairments among patients with IBD and also led to a reduction in the consistency of care during the most acute phases of the pandemic, increasing the disease burden across various groups of patients [13], some of whom also exhibited a decline in therapeutic adherence [14].

Work productivity in IBD and, above all, the predictive factors have been studied heterogeneously without reaching clear conclusions that could provide clinicians with robust tools to identify patients at risk of incapacity or a marked reduction in work productivity. Leso et al. [15], in a systematic review conducted in 2021, identified poorer work pathways for patients with CD due to permanent work cessation. While acknowledging that disease activity can affect work activity, they highlighted the lack of in-depth studies to clarify which symptoms, disease patterns, and parameters are more or less associated with a higher predictive value of work incapacity [15].

As shown by Yousseff et al. [16] in a recent meta-analysis (including studies from Central Europe, Scandinavia, the United States, and Asia), a pooled estimate for absenteeism in IBD was reported at approximately 16.4%.

On the other side of the coin, this work-related unproductivity represents a significant economic burden for societies [17]. From extensive economic analyses filtered for patients with IBD, a five-year follow-up study conducted in the United States on a sample of over forty thousand patients revealed annual direct costs per patient of $24,500, which were significantly higher compared to a non-IBD cohort ($7037 per patient per year) [18]. The peak of such expenses was observed in patients with surgical CD, amounting to $101,013 per patient per year [18]. Hence, it is necessary to understand which factors can reduce healthcare costs related to disability for these patients, ensuring their regular return to everyday life and work productivity. For instance, Zagórowicz et al. [19] analysed expenses incurred by the Polish Social Insurance Institution between 2012 and 2021, demonstrating a decrease in such costs within the IBD population thanks to the use of advanced biological drugs during the time frame [19]. They reported an inverse relationship between the percentage of individuals receiving innovative medicines and the expenses incurred per person across various Polish provinces [19].

This study aims to assess absolute and relative work absenteeism and the corresponding working hours in a group of patients with IBD and to conduct profiling to identify categories of patients more prone to work unproductivity.

## 2. Materials and Methods

### 2.1. Study Design and Setting

This work is an observational cross-sectional survey study conducted from December 2024 to March 2025 at the Division of Hepatogastroenterology of the University of Campania Luigi Vanvitelli, involving the following: (1) consecutive patients with a confirmed diagnosis of IBD [20]; (2) active workers (including active students, involved in equivalent to employment activities); patients with (3) disease activity no greater than moderate, selected using the Harvey–Bradshaw index [21] and the partial Mayo score [22] for inclusion in the study.

Several exclusion criteria were set: (1) unemployed patients; (2) with a still uncertain or differential diagnosis of IBD (e.g., patients with uncertain diagnoses including ischemic colitis, infectious colitis, radiation colitis, and similar conditions); (3) patients with severe psychiatric disorders; (4) patients unfit for work due to prescriptions imposed by occupational health physicians; (5) patients with severe motor disabilities hindering any form of work; (6) hospitalised patients; (7) convalescing patients after surgery; (8) patients with severe clinical disease activity.

All selected patients were sent a link to a completely anonymous questionnaire to collect the variables of interest, ultimately aggregating the data to prevent the traceability of individual patients. The questionnaire could only be submitted once fully completed; therefore, data missing were not considered in data collection. The study was written and presented following the STROBE statement for cross-sectional studies [23].

### 2.2. Questionnaire Structure: Collected Variables

#### 2.2.1. General Section of the Questionnaire

The questionnaire consisted of several sections. The first section collected clinical-demographic data, such as sex, age, body mass index, employment status, educational level, lifestyle habits (smoking, alcohol consumption), type of IBD (CD or UC), possible use of advanced medical therapies (biologics/small molecules), and whether the patient had a stable partner.

#### 2.2.2. Psychometric Assessment Section for Anxiety Levels

In addition, the Beck Anxiety Inventory (BAI) questionnaire [24] was administered, which investigates the severity of anxiety symptoms based on 21 questions (each scored from 0 to 3); specifically, it evaluates the physiological, emotional, and cognitive symptoms of anxiety. According to the BAI, a score of 0–21 indicates mild anxiety, 22–35 indicates moderate anxiety, and scores equal to or above 36 indicate concerning levels of anxiety symptoms [24].

#### 2.2.3. Section Assessing Patient-Reported IBD Disease Activity

IBD disease activity was then assessed using the Patient-Reported Outcome 2 (PRO-2) for CD, evaluating the subscore for the number of bowel movements and that for abdominal pain (rated for severity from 0 to 3 as absent, mild, moderate, or severe, respectively) [25]. To the subscore for bowel movements and that for pain, a multiplier of 2 and 5, respectively, was applied to the weekly average (last 7 days) of the respective daily values, obtaining a total score [25]. Based on data from the previous seven days, remission was identified as a bowel movement frequency subscore ≤ 1.5 and an abdominal pain subscore ≤ 1 [25]. For UC, the PRO-2 involved the bowel movement frequency and rectal bleeding as two subscores, each rated from 0 to 3 based on severity [26]. A total PRO-2 score of 0 identified remission, whereas any other score indicated active disease [26]. The PRO-2 tools are central to the current STRIDE-II guidelines for the assessment of therapeutic targets in the IBD treat-to-target strategy [4].

#### 2.2.4. Section Assessing Work Absenteeism

The assessment of work productivity was conducted using the Health and Work Performance Questionnaire (HPQ) in its short form (© Ronald C. Kessler, PhD), for which authorisation for use was obtained on 3 June 2024 [27,28]. This questionnaire weighs, over a 7-day interval, the number of hours worked (B3) against those expected (B4), as well as the number of full or partial working days lost (due to both health-related and other reasons) and the number of hours worked over a 4-week interval (B6). Additionally, respondents are asked to rate their work performance over the past 1–2 years and the last 4 weeks using a 0–10 Likert scale and compare it to most of their colleagues [27,28]. Relative and absolute absenteeism are then estimated. Within the 7-day interval, absolute absenteeism, defined as the absolute number of working hours lost per month, is estimated as 4 × B4 − 4 × B3 [27,28], while relative absenteeism (i.e., the percentage of expected hours compared to those worked) is calculated as (4 × B4 − 4 × B3)/4 × B4 [27,28]. In the 4-week estimate, absolute absenteeism is calculated as 4 × B4−B6 and relative absenteeism as (4 × B4 − B6)/(4 × B4) [27,28]. Additionally, the HPQ includes three questions that assess, using a 0–10 Likert scale for each, the usual self-perceived performance of colleagues, one’s performance over the past 1–2 years, and one’s performance over the past 4 weeks. Negative values of relative or absolute absenteeism indicate possible overtime work compared to the expected working hours for the time frame in question.

### 2.3. Statistical Analysis

Descriptive statistics were utilised for data presentation, with continuous variables expressed as the median (interquartile range), depending on the normality of the data, which was assessed using the Kolmogorov–Smirnov or Shapiro–Wilk tests, as appropriate (using a sample size cut-off of 50, beyond which the Kolmogorov–Smirnov test was applied). Categorical and ordinal variables were presented as frequencies, reporting the absolute number and the percentage of the total. Qualitative non-continuous variables were compared using either the Chi-square test or Fisher’s exact test, as appropriate. For comparisons involving ordinal and continuous variables, the Mann–Whitney U test or the Kruskal–Wallis test was employed, depending on the degrees of freedom of the grouping variable. A logistic regression model was constructed to assess the impact of work productivity loss in terms of absenteeism on the target variables of interest. This model was evaluated using the Hosmer–Lemeshow goodness-of-fit test, alongside Cox and Snell R^2^ and Nagelkerke R^2^ values, with the results expressed as the exponential value of B [i.e., exp(B)]. These results were also presented as the Odds Ratio (OR), with risk measures reported as the OR along with its 95% confidence interval (95% CI). Furthermore, ORs were adjusted for potential confounding variables (aORs). Spearman’s correlation test was applied where a correlation analysis was required, reporting the correlation coefficient (*ρ*) and the corresponding *p*-value.

All analyses were conducted using a two-tailed significance level, with an alpha error of 5%, defining statistical significance as a *p*-value below 0.05. Statistical analyses were performed using IBM^®^ SPSS^®^ software (version 25, IBM Corp.©, Armonk, NY, USA), while graphing was carried out with Prism GraphPad^®^ (version 9.5.0, GraphPad Software LLC©, Boston, MA, USA). As this was an anonymous cross-sectional survey with consecutive enrolment, no a priori power calculation was established. However, we conducted post-hoc checks to confirm the adequacy of the statistical power using G*Power (version 3.1.9.6, Faul, Erdfelder, Lang, & Buchner, Dusseldorf, Germany), assuming an effect size *d* of 0.4, an alpha error of 0.05, and a power of 0.8.

## 3. Results

### 3.1. Baseline Characteristics of the Sample

A total of 370 patients were initially invited to complete the survey, but 18.9% ultimately did not participate. Of these, 40 (18.81%) did not meet the inclusion criteria, and 30 (8.11%) declined to participate in the survey due to time constraints. A total of 300 patients who had submitted a complete survey were included in the analysis, with a median age of 43.5 years (30–56), predominantly affected by UC (N: 167, 55.7%) and male (N: 162, 54%). As shown in Table 1, the sample was generally homogeneous, except for a higher proportion of university graduates among UC patients (19.8% vs. 14.3%, *p* = 0.015), although employment type was comparable (*p* = 0.163). A higher prevalence of smokers was observed among CD patients [(N: 33, 24.8%) vs. (N: 30, 18%), *p* = 0.0001], along with predictably greater use of a biological agent [(N: 98, 73.7%) vs. (N: 96, 57.5%), *p* = 0.004] and a higher prevalence of previous surgery within the same group [(N: 85, 63.9%) vs. (N: 49, 29.3%), *p* < 0.001]. This was an expected result given the well-known higher prevalence of top-down therapeutic strategies [29], surgical rates [30], and smoking prevalence [31] among patients with CD. 

Across the entire sample, in terms of hours lost, absolute absenteeism over four weeks was recorded at 56 (36–76) hours and 2 (−8–20) hours over seven days. Conversely, relative absenteeism was 0.361 (0.25–0.51) hours over four weeks and 0.012 (−0.05–0.135) hours over seven days. Patients who had lost at least 25% of workable hours in terms of work productivity amounted to 226 (75.3%) of the sample.

### 3.2. Demographic Factors Associated with Reduced Work Productivity

As shown in Table 2, certain clinical–demographic factors were associated with reduced work productivity in absolute absenteeism. The subgroups that demonstrated significantly higher absenteeism across all assessments (absolute and relative) and at all time points (4 weeks and 7 days) were patients with CD compared to those with UC (*p* < 0.001) and those who had undergone previous surgery (*p* < 0.05). Conversely, females exhibited higher absenteeism in the 4-week assessment in absolute terms (*p* = 0.024) and relative terms (*p* = 0.044, Table 2). A similar trend was observed among smokers (*p* < 0.05, Table 2).

Among the anamnesis factors, the assessment of Likert scales of self-perceived work productivity revealed that patients who had undergone previous surgery considered their work performance over the last 1–2 years to be worse compared to those who had never undergone surgery [4 (3–7.25) vs. 6 (3–8), *p* = 0.02], as shown in Table 3.

### 3.3. The Relationship Between Anxiety Symptoms and Work Productivity

**Figure 1:**
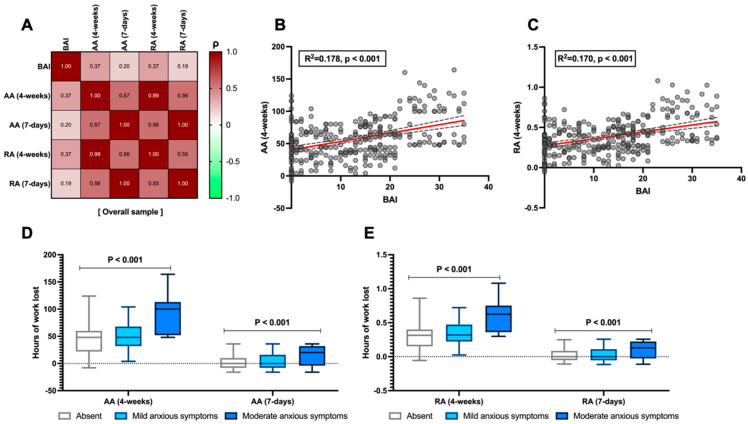
Relationship between the Beck Anxiety Inventory (BAI) and absolute (AA) and relative absenteeism (RA) in the analysed sample. Correlation between the absolute value as the total score of anxious–depressive symptoms measured with the BAI and the levels of AA and RA over both 4-week and 7-day periods (**A**). Linear regression between the BAI and AA (4-weeks) and RA (4-weeks), which showed the highest correlation in the matrix (**B**,**C**), respectively, is also presented. The linear regression line is shown in red, with the corresponding confidence intervals represented as grey dashed lines. Finally, the stratification of the BAI by severity of anxious symptoms is compared to AA (**D**) and RA (**E**) at both time points. According to Spearman’s analysis, the correlation matrices are presented for the overall sample (**A**). Spearman’s coefficient (*ρ*) values are interpreted as follows: >0.69 (robust correlation), 0.40–0.69 (strong correlation), 0.30–0.39 (moderate correlation), 0.20–0.29 (weak correlation), 0.01–0.19 (no correlation). A darker red colour corresponds to a stronger correlation in the graphical representation of the correlation matrix. Continuous variables are illustrated as the median (interquartile range). The *p*-value is also reported to examine differences between the subgroups illustrated in the specific figures, with a significance level set at an alpha error of 5% (with a *p*-value below 0.05).

Anxiety levels, measured using the BAI, demonstrated a moderate correlation, over four weeks, with absolute absenteeism (*ρ* = 0.374, *p* < 0.001, Figure 1A,B) and with relative absenteeism (*ρ* = 0.37, *p* < 0.001, Figure 1A,C). Moreover, this relationship appears to be progressive with the severity of anxiety symptoms. When stratified according to the BAI, the data reveal 42 (14%) patients with no anxiety, 196 (65.3%) with mild anxiety symptoms, and 62 (20.7%) patients with moderate anxiety. As shown in Figure 1D,E, both absolute and relative absenteeism (over 7-day and 4-week periods) progressively increase in terms of work hours lost as the severity of anxiety symptoms rises (*p* < 0.001).

Moreover, as reported in Table 3 (which, as previously mentioned, assessed usual self-perceived work productivity, referring to time frames of 1–2 years and 4 weeks), while the perceived performance of other colleagues remains generally consistent regardless of the severity of anxiety symptoms (*p* = 0.739), conversely, patients’ self-perceived performance progressively declines as the severity increases (*p* = 0.001). The peak of this trend predictably occurs in patients with moderate anxiety symptoms, where the perceived performance of others based on the Likert scale (0–10) is rated at 7 (6–9), whereas their own performance is rated at 3 (2–6.25) when assessed over a 1–2-year period and 3 (1.75–5) when assessed over the past 28 days (*p* = 0.001, Table 3). Moreover, when weighted in logistic regression analysis adjusted for confounding factors such as age, IBD, and sex, the BAI was independently shown to be associated with a 25% loss in work performance in terms of hours not worked out of those expected over four weeks (aOR: 1.1, 95% CI 1.06–1.142, β = 0.096, *p* < 0.001).

### 3.4. The Impact of Disease Activity Assessed Through PRO-2 and Work Productivity

**Figure 2:**
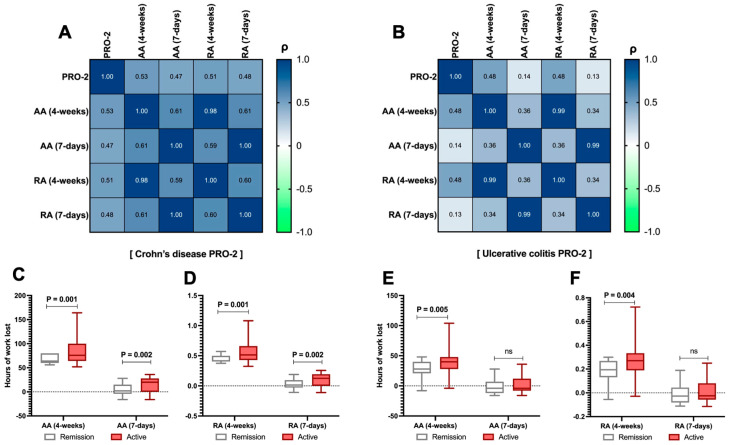
Relationship between the Patient-Reported Outcome 2 (PRO-2) and absolute (AA) and relative absenteeism (RA) in the analysed sample. Correlation between the PRO-2 total score in Crohn’s disease (CD) patients and the levels of AA and RA over both 4-week and 7-day periods (**A**). The same assessment is carried out for PRO-2 in patients with ulcerative colitis (UC, (**B**)). Additionally, by splitting the PRO-2 data (between active/remission UC/CD), the comparison between AA and RA levels was reported at each time point for both CD (**C**,**D**) and UC (**E**,**F**) patients. According to Spearman’s analysis, the correlation matrices are presented for the overall sample (**A**). Spearman’s coefficient (*ρ*) values are interpreted as follows: >0.69 (robust correlation), 0.40–0.69 (strong correlation), 0.30–0.39 (moderate correlation), 0.20–0.29 (weak correlation), 0.01–0.19 (no correlation). A darker red colour corresponds to a stronger correlation in the graphical representation of the correlation matrix. Continuous variables are illustrated as the median (interquartile range). The *p*-value is also reported to examine differences between the subgroups illustrated in the specific figures, with a significance level set at an alpha error of 5% (with a *p*-value below 0.05). *p*-values > 0.05 are identified as “ns”.

From the analysis of disease activity in CD patients, PRO-2 demonstrated a strong correlation with absolute absenteeism (*ρ* = 0.53, *p* < 0.001) and relative absenteeism (*ρ* = 0.51, *p* < 0.001) at 4 weeks, as well as with absolute (*ρ* = 0.47, *p* < 0.001) and relative (*ρ* = 0.48, *p* < 0.001) absenteeism at 7 days, as shown in Figure 2A. Conversely, in UC patients, this correlation was demonstrated only for absolute absenteeism at 4 weeks (*ρ* = 0.48, *p* < 0.001) and 7 days (*ρ* = 0.48, *p* < 0.001), as shown in Figure 2B. Finally, this trend was confirmed when splitting the CD and UC populations into active and remission patients (C-F), considering that patients with active disease were 105 (78.9%) and 143 (85.6%) for CD and UC, respectively. This analysis generally showed, for absolute absenteeism at both time points, a marked difference favouring remission patients regarding work productivity (*p* < 0.01).

Regarding self-perceived performance, patients with active CD reported significantly lower performance compared to those with CD in remission [3 (1.5 − 5) vs. 5 (2.25 − 8), *p* = 0.034], as shown in Table 3. UC did not replicate this finding (*p* > 0.05, Table 3).

As with the BAI, no predictive behaviour of PRO-2 as an independent regression-derived variable for work absenteeism was detected in the multivariate analysis.

### 3.5. Productivity Differences Within the Sample Based on Educational Level and Type of Work Performed

**Figure 3:**
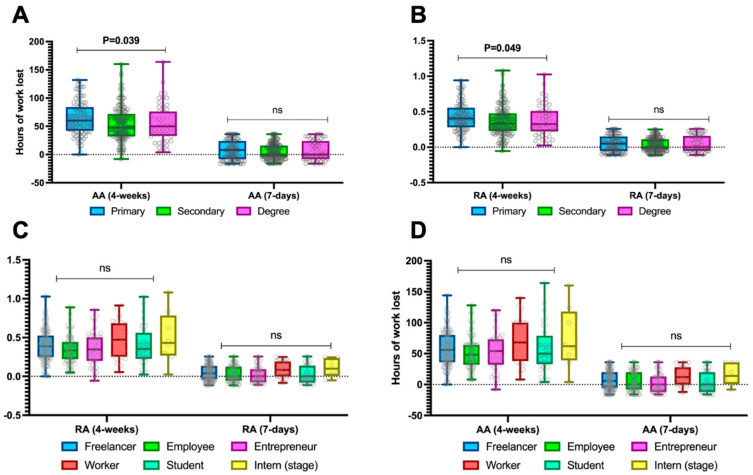
Absolute absenteeism (**A**,**B**) and relative absenteeism (**C**,**D**) stratified by educational level and type of work activity across the entire sample. Continuous variables are illustrated as the median (interquartile range). The *p*-value is also reported to examine differences between the subgroups illustrated in the specific figures, with a significance level set at an alpha error of 5% (with a *p*-value below 0.05). *p*-values > 0.05 are identified as “ns”.

In general, levels of work absenteeism were consistent regardless of the education level or type of work performed (Figure 3), except for a single difference in 4-week relative and absolute absenteeism based on the education level (Figure 3A,B), which showed higher absenteeism among those with primary education compared to those with higher education. Specifically, when considering only 4-week absolute absenteeism, patients with primary education demonstrated values of 60 (42–84) hours lost compared to those with secondary education, who reported 48 (32–72) hours lost, and those with a degree, who reported 50 (33–76) hours lost (*p* = 0.039), as shown in Figure 3A.

**Figure 4:**
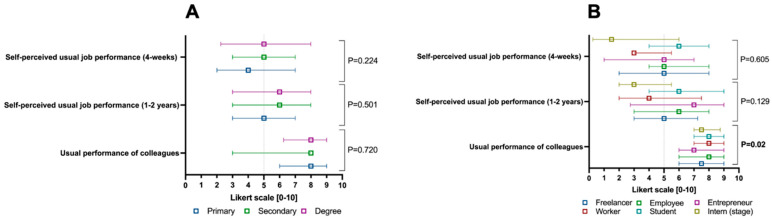
Self-perceived work productivity parameters weighted according to the 0–10 Likert scale of the Health and Work Performance Questionnaire (i.e., HPQ), split by education level (**A**) and macro-category of the work activity performed (**B**). Continuous variables are illustrated as the median (interquartile range). The *p*-value is also reported to examine differences between the subgroups illustrated in the specific figures, with a significance level set at an alpha error of 5% (with a *p*-value below 0.05).

As shown in Figure 4A, the education level did not impact self-perceived work productivity, unlike work activity. For the latter, as illustrated in Figure 4B, there was a difference in the perceived work productivity of other colleagues in the workplace, which was perceived as higher among employed patients, workers, and students compared to other work categories (*p* = 0.02).

## 4. Discussion

In a sample of three hundred patients with IBD engaged in regular work activities, this analysis demonstrated a non-negligible rate of absolute work absenteeism, particularly when considering a time frame set to the last month of work. Indeed, the vast majority (i.e., 75.3%) experience a monthly work productivity loss of at least 25%. From the available meta-analyses, work absenteeism among IBD patients pooled from existing studies is 16.4%, concerning 39.4% weighted pooled work impairment [16].

All this highlights a significant issue in the regular social functioning of patients with IBD [32], who are inherently both subjects and objects of various forms of stigma, including social stigma [33]. The lack of regular social functioning can, in fact, also be a potential cause of social exclusion [34].

Work productivity in patients with IBD has been assessed unevenly across previous studies. In one of the largest studies conducted, Sciberras et al. [35] measured work productivity using the Stanford Presenteeism Scale 6 (SPS-6), demonstrating poor presenteeism in approximately 34% of the sample, significantly lower than our findings. However, this discrepancy may be mainly due to the use of a different assessment tool. Unlike our study, the authors did not identify marked differences between patients with CD and UC. However, they did confirm that patients with anxiety symptoms exhibited poorer work productivity, consistent with our findings. Our data supported poorer work productivity in patients with CD across various measures of absenteeism—whether absolute or relative—and across both weekly and monthly time frames (Table 2). Nasr et al. [36], in a smaller cross-sectional sample of approximately one hundred and sixty IBD patients, found that CD, mainly when presenting with a penetrating phenotype, was an independent factor associated with severe work productivity loss (OR: 6). Other studies have confirmed that patients with CD are more susceptible to work-related disability [15].

Another finding that emerged from our study is a disadvantage in the 4-week absolute and relative absenteeism for females compared to males, with females losing approximately 10 more hours than males, as shown by the median values (Table 2, *p* = 0.024). In our opinion, this further highlights the need for sex-based medicine in IBD, as differences from this perspective have been identified not only in disease epidemiology but also in disease progression and, ultimately, in the response to therapy [37].

As is widely known, disease activity complicates the condition and significantly impairs the quality of life, posing a considerable risk of disability [38]. Our data have demonstrated a clear correlational relationship between disease activity, measured through the PRO-2, and work absenteeism, with predictably higher productivity (and consequently lower absenteeism) observed in patients with disease remission (Figure 2). This finding is more robust as it is not influenced by physician-assessed parameters but rather by the PRO-2 measure, which is directly perceived and reported by patients based on parameters they consider of fundamental importance in their daily lives. Similar results have also been reported in non-European settings, such as in the analysis by Parra et al. [39], which, through a cross-sectional study involving a sample size comparable to ours (407 patients, although not all were employed), demonstrated that moderate-to-severe disease activity was significantly associated with a 75% work productivity impairment compared to those not meeting this criterion (10%). Moreover, Zand et al. [40] also demonstrated that, even during remission, patients with IBD are nonetheless responsible for higher indirect economic costs compared to healthy controls. Moreover, we have also demonstrated that patients undergoing treatment with biologics or small molecules—thus likely presenting with a more complex disease—exhibited similarly higher levels of impairment. This was also true for patients who had undergone surgery, presumably for the same reason (Table 2). Surgery is a well-known factor contributing to disability in IBD [41], and similar studies have already identified it as a threat to work productivity [42].

It is also well known that patients with IBD have a higher prevalence of anxiety compared to healthy controls. A large meta-analysis involving over thirty thousand patients demonstrated that the pooled prevalence reaches 32.1% [43]. Furthermore, in a previous study conducted by our group during the first COVID-19 lockdown in Italy, also using the BAI, we demonstrated that patients with IBD can exhibit significant levels of anxiety, depression, and poor sleep quality even in conditions of complete clinical remission [44].

Our data confirm that anxiety is a significant factor impacting the quality of life of patients, demonstrating a strong correlation with work absenteeism. Moreover, as illustrated in Figure 1, the burden of work absenteeism progressively increases depending on whether absence, mild symptoms, or moderate symptoms are considered, showing a gradual impairment relationship. This type of correlation has also been identified in another study conducted on patients with immune-mediated inflammatory diseases, including 261 with IBD, in which Marcon et al. [45] demonstrated a positive, albeit modest, correlation between anxiety and work productivity loss (r= 0.163; *p*= 0.046), which is nevertheless weaker than that identified in our study. Additionally, in our view, an indirect surrogate of this finding is that, as shown in Table 3, there are also impairments in self-perceived productivity when anxious symptoms, CD, disease activity (particularly in patients with CD), and previous surgery are present. This suggests that there may also be a psychopathological alteration in the patient’s perception of their role within their work environment, which they may perceive as somewhat inadequate compared to the performance of their colleagues.

Additionally, a further consideration that we believe should be carefully examined concerns the differences in the medians of absolute absenteeism over 7 days and 4 weeks, which demonstrate that absenteeism cannot remain constant over time in quantitative terms and undergoes even significant variations within a short period. This is likely attributable to greater intensity of illness activity during certain weekly intervals. In any case, these are speculative considerations that require prospective studies to validate such trends. To this regard, absolute absenteeism measured over 7 days showed a moderate-to-strong correlation with the 28-day estimate (r = 0.63; *p* < 0.001), accounting for approximately 40% of its variance.

This study presents several limitations. Although the study’s cross-sectional nature helps to identify higher-risk subgroups, the absence of a prospective follow-up approach limits the ability to determine whether modifying these risk factors associated with greater work impairment could be amenable to corrective measures (e.g., improving disease activity). Therefore, future research should aim to identify both gastroenterological and psychological/psychiatric strategies capable of addressing intestinal, extra-intestinal, and psychopathological impairment in patients. Additionally, while we have consistently reported sample sizes for each analysis, the statistical power in some subgroup analyses is not always robust, and these specific scenarios should be considered exploratory. Furthermore, despite providing good internal validity, the monocentric nature of the data requires multicentric and international settings to ensure the solid external validity of the parameters identified. Nevertheless, it should be noted that some findings, as previously discussed, have also been observed in settings different from ours, thereby providing reassurance regarding the consistency of the data.

## 5. Conclusions

This cross-sectional study highlighted how work absenteeism in IBD patients may potentially be influenced by various clinical and psychological factors. In particular, patients with CD, female sex, a history of surgical interventions, active smoking, higher disease activity (assessed via PRO-2), and elevated anxiety levels (measured with the BAI) tended to exhibit a higher prevalence of lost working hours and poorer self-perceived productivity (compared to peers). Anxiety emerged as a potential predictor of ≥25% productivity loss in terms of working hours missed, which likely points to the need for further research in this area. These findings underscore the importance of multidisciplinary therapeutic strategies, not only aimed at controlling disease activity but also at identifying and managing psychological comorbidities and other modifiable factors, with the goal of reducing the burden of disability and promoting more effective occupational reintegration for patients. This is in line with the modern concept of disease clearance, which is increasingly emerging as a framework to align the quality of life of these patients with that of the general population.

## Figures and Tables

**Table 1:** Clinical and demographic characteristics of the patient sample included in the survey stratified by type of inflammatory bowel disease (IBD).

Variable	CD N = 133	UC N = 167	*p*-Value ^1^
**Age** (y)	44 (29–55)	43 (32–56)	0.681
**Gender**			0.175 ^3^
Male	66 (49.6%)	96 (57.5%)	
Female	67 (50.4%)	71 (42.5%)	
**BMI** (Kg/m^2^)	27.05 (22.76–32.24)	27.47 (23.72–31.53)	0.543
**Education level**			**0.015**
Primary	53 (39.8%)	40 (24%)	
Secondary	61 (45.9%)	94 (56.3%)	
Degree	19 (14.3%)	33 (19.8%)	
**Job-status**			0.163
Freelancer	62 (46.6%)	56 (33.5%)	
Employee	23 (17.3%)	48 (28.7%)	
Entrepreneur	20 (15%)	26 (15.6%)	
Worker	10 (7.5%)	15 (9%)	
Student	14 (10.5%)	18 (10.8%)	
Intern (stage)	4 (3%)	4 (2.4%)	
**Smoking status**			**0.0001**
Active	33 (24.8%)	30 (18%)	
Past smoker	44 (33.1%)	27 (16.2%)	
Never	56 (42.1%)	110 (65.9%)	
**Alcohol consumer ^2^**			0.460 ^3^
Yes	25 (18.8%)	26 (15.6%)	
**Partner** (yes)	91 (68.4%)	108 (64.7%)	0.495 ^3^
**Biologics** (yes)	98 (73.7%)	96 (57.5%)	**0.004** ^3^
**Steroids active use** (yes)	5 (3.8%)	9 (5.4%)	0.399 ^3^
**Associated arthritis** (yes)	49 (36.8%)	51 (30.5%)	0.250 ^3^
**Diabetes** (yes)	25 (18.8%)	22 (13.2%)	0.183 ^3^
**Previous surgery ^4^** (yes)	85 (63.9%)	49 (29.3%)	**<0.001** ^3^
**Active disease** (yes)	105 (78.9%)	143 (85.6%)	0.700 ^3^

Continuous variables are presented as the median (interquartile range), while categorical or ordinal variables are expressed as frequencies, i.e., counts (percentage of the total in the subgroup considered, CD or UC). Acronyms: CD, Crohn’s disease; UC, ulcerative colitis; BMI, body mass index. ^1^ The test evaluates the differences between the two groups presented (i.e., CD and UC), with an alpha error of 5% and a significance level of a *p*-value < 0.05, with significant values highlighted in bold. ^2^ An individual was classified as an alcohol consumer if their self-reported weekly intake amounted to at least two units of alcohol. ^3^ The Chi-square test (χ^2^) or Fisher’s exact test, as appropriate, was employed in this analysis, with a significance level of *p* < 0.05 for a 5% alpha error. ^4^ Surgery refers to any intervention involving the gastrointestinal and abdominal region.

**Table 2:** Absolute and relative absenteeism values, both over a 28-day (4-week) assessment interval and a 7-day interval, concerning the major subgroups identified within the population sample included in the cross-sectional analysis.

Variable	N	Absolute Absenteeism (4-Weeks)	*p*-Value ^a^	Absolute Absenteeism (7-Days)	*p*-Value ^b^	Relative Absenteeism (4-Weeks)	*p*-Value ^c^	Relative Absenteeism (7-Days)	*p*-Value ^d^
Crohn’s disease	133	76 (64–92) ^#^	**<0.001**	12 (0–28) ^#^	**<0.001**	0.5 (0.4–0.62) ^#^	**<0.001**	0.08 (0–0.19) ^#^	**<0.001**
Ulcerative colitis	167	40 (28–48)	^a^	−4 (−8–12)		0.25 (0.17–0.32)		−0.025 (−0.05–0.075)	
Males	162	50 (32–68)	**0.024**	0 (−8–16)	0.381	0.33 (0.22–0.47)	**0.044**	0 (−0.05–0.11)	0.4
Females	138	60 (40–84) ^#^	^a^	8 (−8–21)		0.4 (0.25–0.55) ^#^		0.05 (−0.05–0.14)	
Alcohol (no)	249	52 (36–76)	0.450	0 (−4–20)	0.574	0.36 (0.25–0.5)	0.559	0 (−0.02–0.135)	0.555
Alcohol (yes)	51	60 (36–80)		4 (−8–20)		0.37 (0.24–0.51)		0.02 (−0.05–0.125)	
Arthritis (no)	200	52 (36–76)	0.246	4 (−7–20)	0.653	0.36 (0.23–0.5)	0.195	0.02 (−0.04–0.132)	0.677
Arthritis (yes)	100	56 (36–80)		0 (−8–20)		0.38 (0.25–0.54)		0 (−0.05–0.135)	
Diabetes (no)	253	52 (36–76)	0.087	0 (−8–20)	0.095	0.35 (0.24–0.49)	0.067	0 (−0.05–0.125)	0.091
Diabetes (yes)	47	64 (36–92)		12 (−4–24)		0.43 (0.25–0.62)		0.08 (−0.02–0.171)	
Smoking (no)	237	52 (36–76)	**0.036**	0 (−8–20)	0.519	0.35 (0.24–0.5)	**0.04**	0 (−0.05–0.138)	0.549
Smoking (yes)	63	60 (40–80) ^#^		8 (−4–16)		0.4 (0.27–0.52) ^#^		0.05 (−0.02–0.114)	
Partner (no)	101	52 (34–78)	0.736	0 (−12–20)	0.407	0.36 (0.22–0.5)	0.684	0 (−0.75–0.142)	0.427
Partner (yes)	199	56 (36–76)		4 (−4–20)		0.37 (0.25–0.51)		0.028 (−0.02–0.125)	
Biologic/SM (no)	106	46 (32–76)	**0.016**	0 (−8–20)	0.904	0.3 (0.2–0.5)	**0.024**	0 (−0.05–0.13)	0.845
Biologic/SM (yes)	194	60 (40–77) ^#^		4 (−8–20)		0.4 (0.266–0.51) ^#^		0.02 (−0.05–0.13)	
No surgery	166	48 (32–64)	**<0.001**	0 (−8–16)	**0.011**	0.3 (0.21–0.43)	**<0.001**	0 (−0.05–0.11)	**0.014**
Previous surgery	134	64 (44–88) ^#^		8 (−4–24) ^#^		0.42 (0.28–0.58) ^#^		0.05 (−0.02–0.15) ^#^	

Notes: continuous variables are presented as the median (interquartile range). N: sample size. SMs: small molecules. The additional subgroups identifiable within the sample but with reduced numbers were not included in the table due to an imbalance in statistical analyses and data instability in interpretative terms. ^a^ The test evaluates the differences between the two subgroups presented in the row in terms of absolute absenteeism assessed over 4 weeks (28 days), with an alpha error of 5% and a significance level of *p*-value < 0.05, with significant values highlighted in bold. ^b^ The test evaluates the differences between the two subgroups in the row regarding absolute absenteeism assessed over 7 days, with an alpha error of 5% and a significance level of *p*-value < 0.05, with significant values highlighted in bold. ^c^ The test evaluates the differences between the two subgroups in the row regarding relative absenteeism assessed over 4 weeks (28 days), with an alpha error of 5% and a significance level of *p*-value < 0.05, with significant values highlighted in bold. ^d^ The test evaluates the differences between the two subgroups in the row regarding relative absenteeism assessed over 7 days, with an alpha error of 5% and a significance level of *p*-value < 0.05, with significant values highlighted in bold. ^#^ In the comparison, this subgroup appears to be the most disadvantaged regarding workplace absenteeism, with a more significant impact on work productivity. This symbol is used for easier identification.

**Table 3:** Self-perceived work productivity scales of the Health and Work Performance Questionnaire (i.e., HPQ) assessed in the main subgroups identified within the sampled population.

Variable	N	Usual Performance of Colleagues [0–10]	*p*-Value ^a^	Self-Perceived Usual Job Performance (1–2 Years) [0–10]	*p*-Value ^a^	Self-Perceived Usual Job Performance (4-Weeks) [0–10]	*p*-Value ^a^
Crohn’s disease	133	8 (6–9)	0.625	4 (2–7)	**<0.001**	3 (2–6)	**<0.001**
Ulcerative colitis	167	8 (6–9)		6 (4–9)		6 (3–8)	
Males	162	8 (6–9)	0.225	6 (3–8)	0.116	5 (2–8)	0.397
Females	138	8 (6.75–9)		5 (3–8)		5 (2–7)	
Alcohol (no)	249	8 (6–9)	0.791	6 (3–8)	0.989	5 (2–8)	0.481
Alcohol (yes)	51	7 (7–9)		5 (3–8)		5 (2–7)	
Arthritis (no)	200	8 (6–9)	0.924	5 (3–8)	0.766	5 (2–7)	0.769
Arthritis (yes)	100	8 (6–9)		6 (3–7)		5 (2–8)	
Diabetes (no)	253	8 (6–9)	0.569	6 (3–8)	0.087	5 (2–8)	0.083
Diabetes (yes)	47	8 (6–9)		4 (2–8)		4 (2–6)	
Smoking (no)	237	8 (6–9)	0.207	6 (3–8)	0.356	5 (2–7)	0.355
Smoking (yes)	63	8 (6–9)		5 (2–8)		5 (2–7)	
Partner (no)	101	8 (7–9)	0.225	6 (3–8)	0.116	5 (2–7)	0.397
Partner (yes)	199	8 (6–9)		5 (3–8)		5 (2–8)	
Biologic/SM (no)	106	8 (6–9)	0.836	6 (3–9)	0.659	5 (2–8)	0.971
Biologic/SM (yes)	194	8 (6–9)		5 (3–8)		5 (2–7)	
No surgery	166	8 (6–9)	0.698	6 (3–8)	**0.02**	5 (3–8)	0.698
Previous surgery	134	8 (6–9)		4 (3–7.25) ^#^		4 (2–7)	
No anxiety	42	8 (6–9)	0.739	6 (4–8)	**0.003**	6 (4–9)	**0.001**
Mild anxious symptoms	196	8 (6–9)		6 (3–8)		5 (2–8)	
Moderate anxious symptoms	62	7 (6–9)		3 (2–6.25) ^#^		3 (1.75–5)^#^	
Crohn’s disease (remission)	28	8 (7–9)	0.472	5.5 (3–8)	0.094	5 (2.25–8)	**0.034**
Crohn’s disease (active)	105	8 (6–9)		4 (2–6)		3 (1.5–5) ^#^	
Ulcerative colitis (remission)	143	8 (6–9)	0.662	6 (3–9)	0.197	6 (3–8)	0.539
Ulcerative colitis (active)	24	8 (6–10)		8 (4.25–9)		5 (4.25–8.75)	

Notes: continuous variables are presented as the median (interquartile range). N: sample size. SMs: small molecules. The additional subgroups identifiable within the sample but with reduced numbers were not included in the table due to an imbalance in statistical analyses and data instability in interpretative terms. ^a^ The test evaluates the differences between the two subgroups presented in the row, with an alpha error of 5% and a significance level of *p*-value < 0.05, with significant values highlighted in bold. ^#^ In the comparison, this subgroup appears to be the most disadvantaged regarding the Likert scale. This symbol is used for easier identification.

## Data Availability

The original contributions presented in the study are included in the article, and further inquiries can be directed to the corresponding author.
